# Retroactive Signaling in Short Signaling Pathways

**DOI:** 10.1371/journal.pone.0040806

**Published:** 2012-07-26

**Authors:** Jacques-Alexandre Sepulchre, Sofía D. Merajver, Alejandra C. Ventura

**Affiliations:** 1 Institut Non Linéaire de Nice, UMR 7335 CNRS - University of Nice Sophia Antipolis, Valbonne, France; 2 Department of Internal Medicine, and Comprehensive Cancer Center, University of Michigan, Ann Arbor, Michigan, United States of America; 3 Instituto de Fisiología, Biología Molecular y Neurociencias, CONICET and Departamento de Fisiología, Biología Molecular y Celular, Universidad de Buenos Aires, Buenos Aires, Argentina; National Cancer Center, Japan

## Abstract

In biochemical signaling pathways without explicit feedback connections, the core signal transduction is usually described as a one-way communication, going from upstream to downstream in a feedforward chain or network of covalent modification cycles. In this paper we explore the possibility of a new type of signaling called retroactive signaling, offered by the recently demonstrated property of retroactivity in signaling cascades. The possibility of retroactive signaling is analysed in the simplest case of the stationary states of a bicyclic cascade of signaling cycles. In this case, we work out the conditions for which variables of the upstream cycle are affected by a change of the total amount of protein in the downstream cycle, or by a variation of the phosphatase deactivating the same protein. Particularly, we predict the characteristic ranges of the downstream protein, or of the downstream phosphatase, for which a retroactive effect can be observed on the upstream cycle variables. Next, we extend the possibility of retroactive signaling in short but nonlinear signaling pathways involving a few covalent modification cycles.

## Introduction

One of the most vital processes in biology is the transduction of signals along biochemical pathways, enabling the living cell to elicit appropriate responses to chemical and physical stimuli [1]. In this context, the concept of signaling cascade is used as a paradigm or a model of signaling pathways. It consists of a chain of enzymatic reactions wherein a protein is interconverted reversibly between two forms. At each stage in the cascade, the activated form of the protein, which usually is a covalently modified derivative of the native protein, serves as the enzyme to activate the protein in the next stage in the chain and so on. Thus, a signaling cascade consists of a succession of covalent modification cycles, whose classical representative example is the phosphorylation/dephosphorylation cycle, but the general concept is broadly applicable. In some important cases, such as the well-studied MAPK cascades, the stages are in fact composed of double phosphorylations [2], [3]. In all cases, the concept of cascade clearly indicates a notion of flow oriented unidirectionally.

A general intracellular signaling network may consist of several interconnected cascades [4]. Its topology can then be described as an oriented graph whose nodes represent stages of the cascades and the arrows serve to relate the activated proteins at a given stage to other covalent modification cycles or to a substrate targeted by the network. Associated with such a graph one may define several signaling pathways, namely several paths in the oriented graph, going from a top vertex, representing a biochemical entry of the system, e.g. a ligand, towards the bottom stage of one of the cascades, e.g. a transcription factor for some genes. A simple type of signal that can be transmitted in this system is a step increase of the enzyme activating the top cycle of one signaling pathway. Several studies have been devoted to the modeling of the propagation of such signal in signaling chains, and on the transmission properties as a function of most of the parameters of the cascade [3], [5]–[7].

The mathematical modeling of signaling pathways often considers a simplified set of equations in which each cycle is described by a single variable [5]. In a previous study, we highlighted that these simplified models overlooked the property of retroactivity between two successive stages of the cascades, and we proposed a new type of simplified modeling for cascades to account for this important signaling property [8]. The concept of retroactivity means that the response property of a well-characterized input/output isolated device can change dramatically when this device is coupled to a downstream load. In the context of signaling pathways, retroactivity is a phenomenon that arises due to enzyme sequestration in the intermediate complex enzyme-next protein in the cascade. Its main consequence is that a downstream perturbation -e.g. of the protein- can produce a response in a component upstream of the perturbation without the need for explicit feedback connections. In refs. [8], [9] this effect was described independently by two groups for the first time. The main focus in ref. [8] was to derive a simplified description of signaling cascades with one variable per cycle while keeping the retroactive property, after noticing that the standard simplifications on modeling cascades were explicitly avoiding such effects. The study of the effect (referred to as retroactivity in [9]) was done mostly numerically in [8], introducing the notion of “reverse stimulus response curve”. Now, we study in detail reverse stimulus response curves, by characterizing both analytically and numerically when to expect a measurable upstream effect due to a downstream change in a control parameter. This work provides a roadmap for planning experiments that carefully account for this phenomena.

The absence of retroactivity for a signaling module implies that the state variables of this module do not change when its output is used as the input of another device. Special conditions are to be met in the design of a network unit in order to minimize the retroactivity [9], [10]. In the context of engineering, and specifically in synthetic biology where modularity is required [11]–[13], retroactivity is usually considered as a nuisance, often preventing the proper functioning of devices that consists of assemblies. The property of pathway retroactivity started to gain interest in the systems biology community [9], [14]–[16]. Retroactivity tends to be attenuated in long signaling cascades [7], [10]. However, ref. [10] also shows that the probability that a 3-stage cascade exhibits retroactivity is around 0.5, so under many commonly encountered conditions, retroactivity occurs. Indeed, recent experiments demonstrate that retroactivity can be set in evidence and measured *in vivo* in the MAPK cascade controlling the early development of drosophila embryos [17]. An *in vitro* study shows that retroactivity effects can be easily induced at one stage of the signaling system regulating the nitrogen assimilation in *E. coli* [18]. In short, retroactivity can be experimentally demonstrated in signaling pathways. In the recent paper by Wynn et al [16], it is shown that an important consequence of retroactivity is its role in the cellular response to a targeted therapy. In particular, we characterized the fact that kinase inhibitors can produce off-target effects as a consequence of retroactivity. In this numerical study, a statistical methodology based on a random sampling of the parameter space was utilized. In particular, that study considered a signaling topology with 3 single cycles, where one of them activates the other two in parallel. This system is also analysed in the present paper which is based on a numerical and analytical study of the nonlinear equations. In that sense, both articles complement each other.

**Figure 1:**
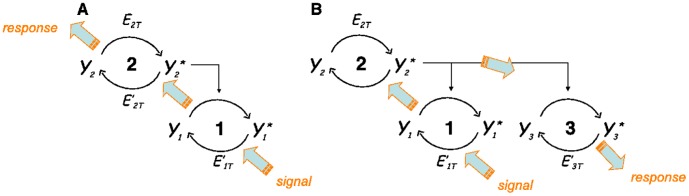
Motifs of short signaling pathways illustrating the concept of retroactive signaling in (A) a 2-cycle cascade and (B) in a 3-cycle cascade. Thick arrows indicate the direction of signaling.

Moreover, in the present work, we make use of the property of retroactivity in order to extend, theoretically, the standard view of signaling to a new type of intracellular signaling. Indeed, the existence of retroactivity in signaling pathways turns the usually one-way oriented graphs mentioned above, into two-way oriented graphs, with arrows going now from downstream to upstream. We call *retroactive signaling* the design of a pathway that exploits this possibility, that is to say, an extended signaling pathway which comprises a connected path of upstream signaling from output to input (cf. Fig. 1). Since retroactivity is a secondary effect, when compared with the usual activation in signaling cascades, a retroactive signaling pathway would typically include only one or a few upstream arrows combined with the usual downstream arrows. Nevertheless, the possibility of retroactive steps in a signaling pathway opens up previously unexplored possibilities for signal transduction.

In this paper we explore this concept for the first time in short signaling pathways like the basic case of a 2-cycle cascade and simple extensions of it. The 2-cycle cascade, or the bi-cyclic cascade, is usually described as a motif comprising 2 cycles and a single arrow linking the activated protein of the first onto the second cycle. In this article, retroactive signaling in this system will be dealt with by analysing how a variation of the parameters affecting the downstream cycle, e.g. varying the total protein concentration in this cycle, or its phosphatase, can induce a response in variables of the upstream cycle. The upstream response can be computed numerically and estimated analytically. We will illustrate the theoretical work with examples of retroactive signaling in short multi-cycle pathways.

## Results

### The Main Question

Figure 1 depicts simple motifs of 2-cycle and 3-cycle pathways. The goal is to study the conditions under which a signal, or a perturbation, that modifies the state of a *downstream* cycle, can be transmitted *upstream*, to another cycle in the context of these short pathways. We will focus most of our studies on what happens to the upstream cycle in a 2-cycle system, when control parameters of the downstream cycle are modified, as for instance its total available protein or its total phosphatase.

The mathematical equations describing these systems are discussed in the Methods section. To summarize our main notations, we name each cycle in a given signaling pathway by an index *i*(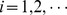). We take the convention to call cycle 1 the starting cycle of a retroactive signaling scheme, and to increment the number of the other cycles following their position in the signaling network until the last cycle in the pathway has been reached. Figure 1(B) shows a simple example of retroactive signaling in the pathway 1
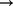
2
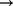
3 where cycle 2 is an enzyme for both cycles 1 and 3. For notational convenience we will use variable names to denote both a chemical species and its concentration. For instance, the instantaneous state of each cycle is described by the variables 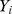 and 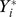, denoting respectively the concentrations of the inactivated and of the activated protein 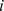, whose total amount is denoted by 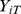. The enzymatic activations of a given stage of the cascade on the next stages are indicated by vertical top-down arrows on Fig. 1, except for the activation of the uppermost stage for which the activating enzyme is a parameter, e.g. 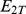 denoting the total concentration of the enzyme converting 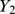 into 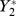. In all cases, the enzyme deactivating cycle 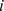 has a total concentration denoted by 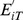.

In most signaling systems, the activated form of protein *i* corresponds to its phosphorylated form, in which case the converting enzymes are called *kinase* and *phosphatase*, respectively for the phosphorylation and the de-phosphorylation of the protein. Since this situation is the most frequently present in intracellular signaling modules, in what follows we will often name 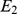 the kinase and 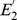 the phosphatase of cycle 2, just for brevity. Moreover, the activating covalent modification will be referred to as phosphorylation. In fact, all the formalism used in this study can equally well apply to other covalent modifications like adenlylation, methylation, GTP-ase modifications.

### Varying the Available Protein in a Signaling Cycle

In order to describe the 2-cycle cascade (cf. Fig. 1(A)) from the point of view of retroactive signaling, let us start by suppressing the phosphatase in the upstream cycle, i.e. set 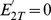 in cycle 2. Then, cycle 1 behaves like a single signaling cycle with kinase 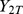 and with phosphatase 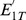. Let us analyse what happens to the activated and the non-activated proteins in cycle 1, when the total available amount of this protein, denoted by 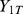, is varied between 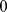 and an arbitrarily large value. In what follows, we will see that answering this question will provide a way to analyse simple instances of retroactive signaling.

**Figure 2:**
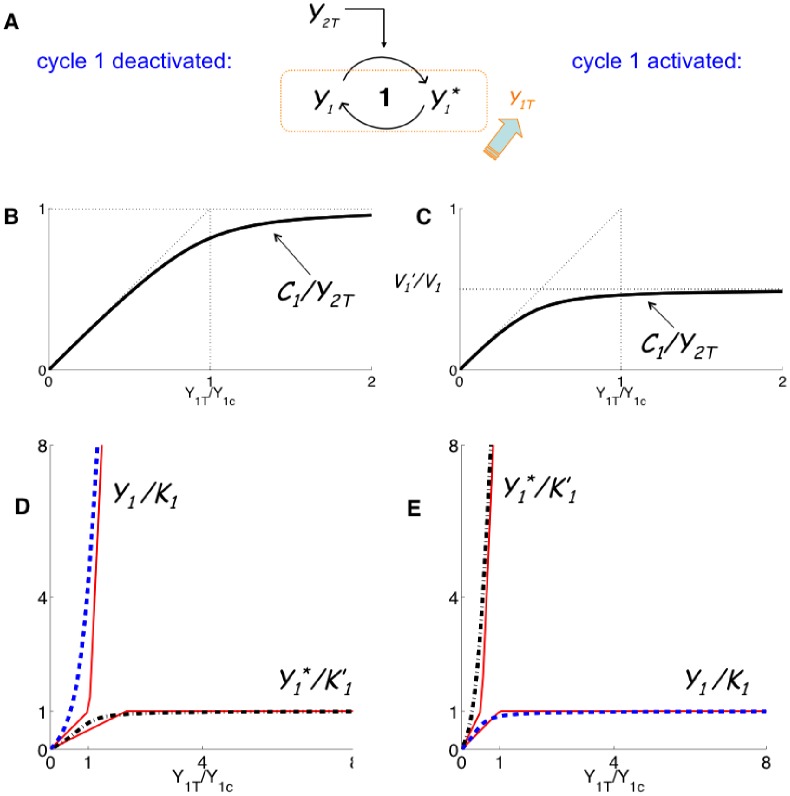
Behaviors of cycle 1 as a function of 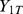, the total protein in cycle 1. The kinase for this cycle is denoted by 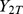 and the phosphatase by 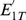. The abscissa are scaled by the characteristic range 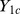, cf. Eq. (1). A) Two cases are considered for cycle 1, which is said deactivated if 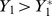 and activated if 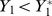. B-C) Increase of the intermediate complex 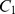 when cycle 1 is respectively deactivated or activated. D-E) Variations of activated 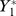 and non-activated 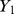 proteins in the two cases 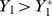 and 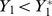. The graphs were obtained by solving Eqs.(16)-(18) with the following parameters : 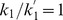, 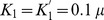
M, 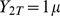
M, 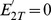; panels (B-D) : 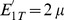
M.; panels (C-E) : 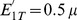
M.

The intermediate complex 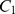 formed by enzyme 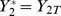 and protein 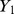 is a key chemical species in the coupling between cycle 2 and cycle 1. Thus it is relevant to study how 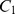 grows when the total protein of cycle 1 is increased from the value 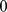. Figure 2(B) shows the case where cycle 1 is deactivated (*i.e.* 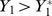). Then, 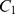 first increases proportionally to 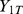, and reaches a plateau corresponding to its saturated value, 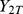, when 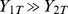. This saturating behavior suggests the definition of a characteristic range for the variation of 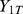, meaning that above this range a further increase of total protein in cycle 1 has not much effect on the sequestration of protein in cycle 2. For example, we can define the characteristic range for 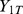 by extrapolating the initially linear growth of 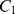 as a function of 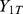 to its asymptotic value 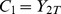. This is indicated and denoted on Fig. 2 by 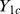. This characteristic range of 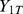 can be analytically calculated as a function of the parameters of cycle 1. The result is:
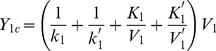
(1)where 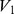 and 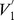 are the maximal reaction rates defined in Eq.(19), and 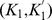 are the Michaelis-Menten coefficients of the cycle 1 (cf. section Methods). The quantity 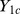 will be used in the following in order to non-dimensionalize the parameter 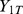 by scaling it with 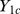 whenever 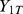 is plotted (e.g. in abscissa).

Figure 2(C) shows the increase of 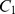 when cycle 1 is activated (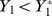). It can be shown that in this case the maximum amount for 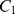 is 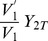, with 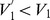, meaning that the sequestration is lower than in the case where cycle 1 is deactivated. Therefore, we will see in the next Section that in order to optimize the retroactivity in a 2-cycle system, the downstream cycle should be deactivated, so that varying 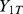 has a larger effect on 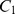 and thereby a greater influence on the upstream cycle.

At the same time, two distinct behaviors are seen for variables (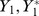) as a function of total 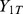, according to whether cycle 1 is activated or not (cf. Fig. 2(D-E)). If cycle 1 is deactivated the asymptotic behavior is a linear increase of variable 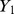 while 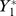 tends to a constant. If cycle 1 is activated, the converse happens, namely 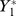 grows linearly and 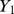 reaches a constant value. Therefore, increasing the amount of substrate 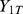 beyond the characteristic range 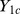 in the covalent modification cycle 1 tends to an increase of either the activated or of the deactivated protein, but not of both, and the other variable tends to a constant. These latter values can be computed analytically as follows, if 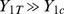 (cf. the section Methods):

- if 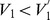 then 
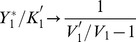
(2)
- if 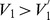 then 
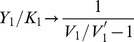
(3)

Figures 2(D-E) illustrates also that the graphs of 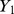 and 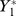 as a function of 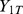 can be sketched by piecewise linear approximations. In particular, the initial slope of 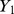 with respect to 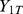 is found to be 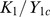, whereas the initial slope of 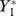 is 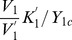 (cf. section Methods).

The results of this section were obtained by assuming absence of phosphatase in cycle 2, so that cycle 1 behaved as an isolated cycle. In the general case of a 2-cycle system, with some phosphatase acting in the upstream cycle (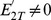), the obtained results can change, but the modifications are worked out in the Method section. Particularly, one shows that the characteristic range for 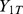, which are now denoted by 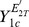, has a similar expression to the one defined by Eq.(1), but replacing in this equation 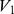 by 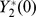, where 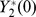 is the phosphorylated protein in cycle 2, in the limit of vanishing 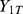. Nevertheless, it appears that 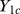 (Eq.1) is useful as an upper bound of the characteristic range 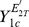, whose a lower bound is given by 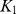. Regarding the behavior of the cycle when 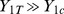, Eq.(2) still holds whatever the value of 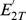 is, if 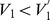. On the other hand, when 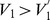 and 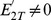, the limit (3) gives the final value of 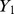 only approximately. The exact asymptotic behavior of 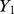, which cannot be formulated as a simple analytical expression, is given in the Method section (cf. Eq.(39)).

### Retroactive Signaling in a 2-cycle Cascade

Having gained insight into how a covalent modification cycle behaves when its total protein 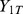 is varied, we ask how the cycle 2, which is upstream with respect to cycle 1, can be influenced by varying parameters of the downstream cycle. In an experimental setup, the downstream cycle 1 can be characterized by 2 control parameters, namely the total protein 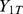 as seen before, and the amount of phosphatase acting on the deactivation of cycle 1, i.e. 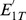. In this section the considered control parameters of the 2-cycle cascade will be 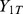 or 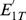.

What kind of variables can we measure on the upstream cycle to observe the effect of varying the control parameters of the downstream cycle? One possibility is to measure the fraction of activated (e.g. phosphorylated) protein in cycle 2 [17]. The latter is defined by:
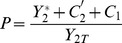
(4)Indeed the intermediate complexes 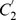 and 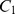 both contain some fraction of the phosphorylated protein in cycle 2. In particular, 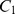 represents the fraction of activated protein 2 that is sequestered in cycle 1. Thus this variable embodies the coupling between the two cycles and the source of retroactivity.

**Figure 3:**
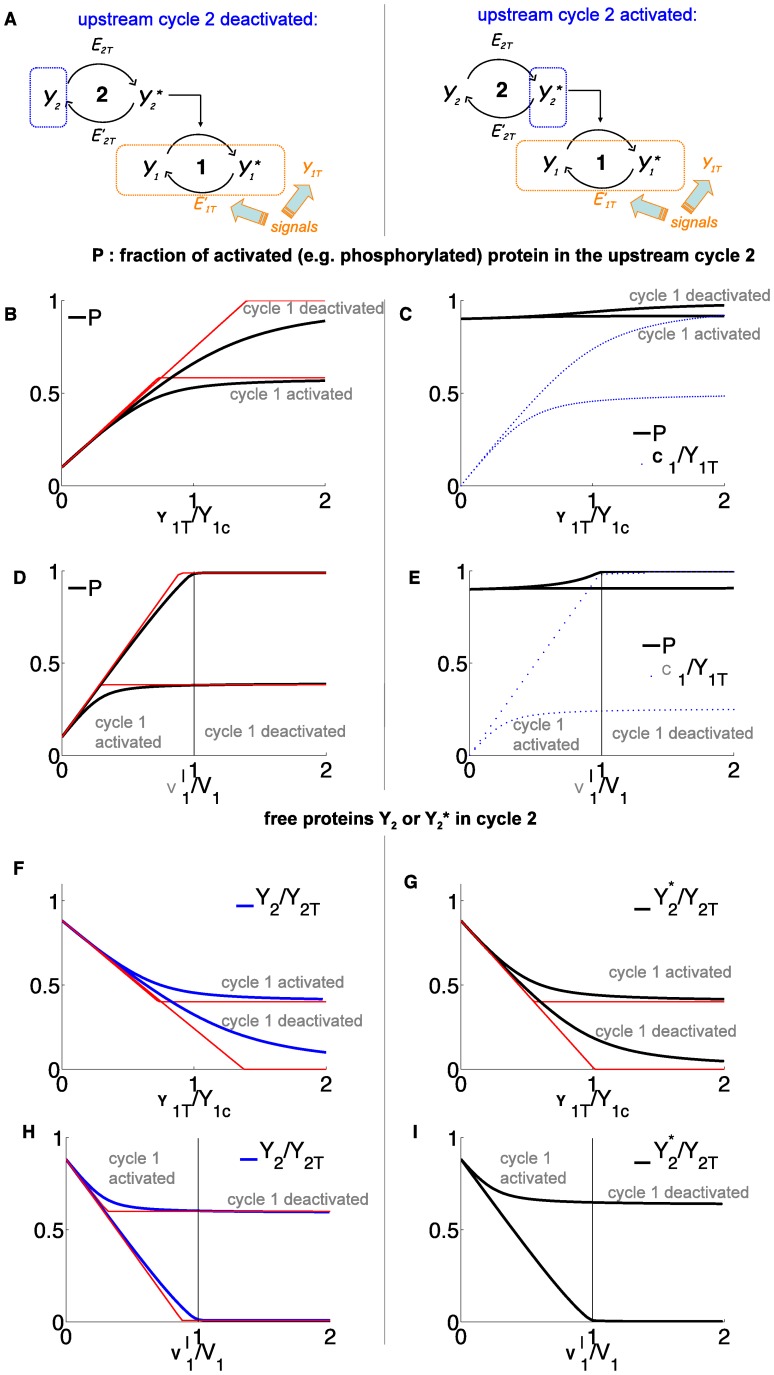
Phosphorylated fraction of protein 2 as a function of 2 control parameters of the downstream cycle 1, namely 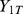 and 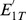. The graphs are obtained by solving Eqs.(16)-(18) with the following parameters : 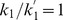, 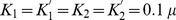
M, 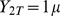
M; On the left figures (B,D,F,H) cycle 2 is assumed deactivated, with 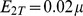
M
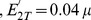
M. These values are swapped for the right figures (C,E,G,I) where cycle 2 is assumed activated. Panels (B,C,F,G) : cycle 1 is either deactivated (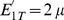
M), or activated (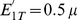
M). On panels (D,E,H,I), phosphatase 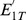 is varied from 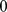 to 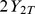 (so that 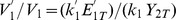 varies from 0 to 2). Panels (D,H) : for the upper curve the total protein 1 is 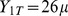
M and for the lower curve 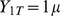
M. Panel (E,I) : for the upper curve the total protein 1 is 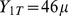
M and for the lower curve 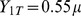
M.

Figure 3 shows the variations of the activated fraction 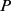 as a function of parameters 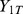 and 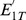 under several conditions, depending on cycle 2 is activated or not. As will become clearer in the next sections, the main message of Fig. 3 is that varying the downstream parameters, the retroactivity on the phosphorylated fraction 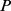 is significant only when the upstream cycle starts in deactivated state (left column). It is relatively negligible however, when the upstream cycle starts out activated.

#### Varying the available protein of the downstream cycle

Let us consider in detail the effect of varying the total protein 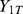 in cycle 1. In practice, this can be achieved in various ways, e.g. by overexpressing the gene coding for protein 1, or by interfering with this quantity by adding a drug able to inhibit this protein [16], or by sequestration of 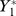 resulting from modifying its substrates [19]. Since the retroactive control of cycle 1 on cycle 2 depends crucially on the complex 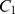, the relevant range of variation for 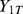 can be estimated by 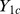 given by Eq.(1). Therefore, the graphs presented in Figs. 3(B-C) show variations of 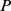 over a range of 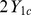, which is adequate to capture the significant variations of the activated fraction of protein 2 induced by varying 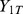. Figure 3(B) shows that when cycle 2 is deactivated, the variation of 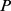 can pass from a value close to 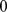 to a value close to 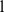. Moreover the amplitude variation of 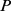 is maximum when cycle 1 is deactivated. In the latter case, we have seen in the previous section that the non-activated protein 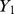 grows proportionally to 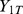 (Fig. 2(D)). This arbitrarily large increase of the substrate of 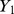 causes the saturation of enzyme 1 for cycle 1 and the complex 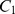 increases towards its maximal allowed value 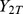 like in Fig. 2(B). Therefore, by increasing 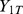, the phosphorylated fraction 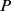 tends to its maximal value 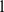; in this case we have a phenomenon of *total sequestration* of protein 2 in cycle 1.

On the other hand, if cycle 1 is activated and cycle 2 is still deactivated, the results of the previous section show that 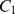 reaches only a fraction of total protein 2, namely 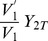 (Fig. 2(C)). Here we observe a phenomenon of *partial sequestration* of species 2 by cycle 1. Once this partial sequestration has occurred, a further increase of 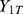 has no longer an effect on the upstream cycle 2. The latter behaves then as a single covalent modification cycle with a reduced amount of protein 2, equal to 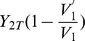. Therefore, the fraction 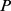 saturates sooner than before and remains inferior to 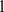. It is seen on Fig. 3(B) (thin red lines) that a piecewise-linear sketch for the variations of 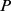 is sufficient to describe the behavior of 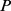 as a function of 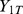.

Finally, the case where cycle 2 starts out activated is depicted on Fig. 3(C). In this situation, the phosphorylated fraction 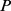 hardly varies whatever the value of 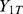 is, especially if cycle 1 starts out also activated. If it is deactivated, the variation of 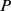 is non zero, but very weak. In conclusion, in order to enhance the retroactive control of cycle 1 on cycle 2, that is to get the larger possible increase of the fraction of phosphorylated protein in cycle 2, and this as a function of parameter 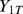 of cycle 1, one should start from a situation where both cycles 1 and 2 are deactivated.

#### Varying the phosphatase of the downstream cycle

We turn now to the retroactive effect of varying the phosphatase of the downstream cycle, 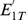, on the fraction of phosphorylated protein in cycle 2. Here the total protein 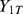 is fixed. Figures 3(D-E) show the variation of the phosphorylated fraction 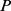 as a function of 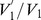, that is a non-dimensionalized parameter proportional to 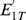 (Eq.(19)). In the same manner as before, one observes that the phosphorylated fraction 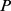 exhibits a significative variation only in the case where cycle 2 is deactivated (Fig. 3(D)). Moreover, the variation of 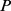 is seen only when the control parameter 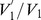 varies in the interval 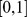, that is when cycle 1 passes from its activated to its deactivated state. Then, the level of 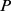 increases proportionally to 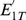, until reaching a plateau depending on the chosen amount of 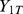. This plateau, that is the maximum fraction of upstream protein 2 that can be phosphorylated by increasing the phosphatase of the downstream cycle, can be predicted by the expression:
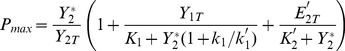
(5)This equation is derived below, in the section Methods. In this equation, 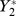 is the maximum free protein 2 that is activated in the limit of arbitrarily large phosphatase 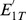. Thus it is unknown a priori but, as a first approximation, it can be replaced by 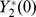 (the value of 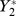 in absence of cycle 1). To get a better estimate, the actual value of 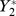 can be found by using an iterative process.

Equation (5) allows us to estimate the level of 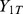 necessary to reach a given fraction 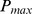 in the limit of large phosphatase 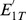:
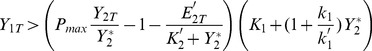
(6)


In summary, in a 2-cycle cascade, in order to create conditions that may substantially modify the fraction of the activated protein in the upstream cycle by perturbing the parameters of the downstream cycle, it is recommended to deactivate the upstream cycle 2. Then, if the downstream cycle 1 is also maintained deactivated a substantial change in 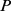 can be obtained by varying the total protein in the downstream cycle, within a range 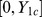, where 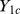 can be computed as a function of the system parameters (Eq.(1)). In the case where the downstream cycle is activated, it is also possible to change 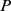 by varying the total protein, but in a smaller range than before, namely 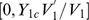. Varying the phosphatase of the downstream cycle will not modify 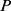, if cycles 1 and 2 are both deactivated. If, on the other hand, the downstream cycle is activated, then a retroactive signaling in 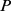 can be achieved by modifying the downstream phosphatase, provided that the total protein 1 is sufficiently abundant (cf. Eq.(6)).

The above analysis focussed on the changes of the fraction of phosphorylated protein in cycle 2 because the variable 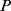 is experimentally accessible. However, it is also interesting to describe the behaviors of the 2-cycle cascade in terms of the free proteins in cycle 2, respectively 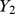 and 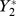, as will be covered in the next section. Indeed, as discussed below, 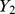 and 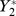 are responsible for the possible crosstalk effects in cascades with more than 2 cycles.

#### Downregulation of the free proteins in the upstream cycle

When the upstream cycle 2 is deactivated, Figs. 3(B,D) demonstrate that the phosphorylated fraction 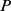 can be raised by increasing 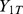 or 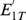 from 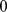. How does this growth affect the amount of free non-active and active proteins in the upstream cycle? It is seen on Fig. 3(F) and (H) that the growth of 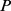 coincides with a decrease of the non-active protein 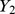. Conversely, the variation of the free activated protein 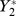 is negligible (not shown). Moreover, if cycle 1 is deactivated, addition of the substrate 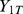 in cycle 1 can lead to a complete depletion of protein 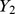 in the upstream cycle. The decrease of 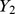 is roughly linear in the range 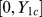, and then beyond this range it is inversely proportional, 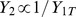. When the downstream cycle is activated, the decrease of 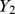 occurs on the smaller range 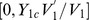 and then reaches a plateau that can be analytically predicted (cf. thin continuous lines on Fig. 3)(F)). This situation reflects the phenomenon of partial sequestration of protein of cycle 2 in the dynamics of cycle 1.

As illustrated on Fig. 3(H), the variation of phosphatase in the downstream cycle can also retroactively affects the amount of non-activated protein 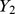, provided that cycle 1 is activated and that the quantity 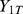 is large enough. This figure also shows that the variation of 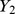 is well approximated by a linear decrease as a function of 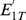 or, equivalently, of 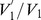.

When the upstream cycle 2 is activated, Figs. 3(C,E) showed that a variation of control parameters in cycle 1 entailed only minor changes in the fraction of phosphorylated protein in the upstream cycle 2. This result might convey the idea that when cycle 2 is activated no retroactivity can be observed on cycle 2. In reality, this view would be wrong, because in this case there can exist a large decrease of the free active enzyme 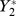, as illustrated on Figs. 3(G,I). Indeed, although the fraction 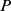 stayed relatively constant on Figs. 3(C,E), these graphs showed also that the amount of protein 2 sequestrated by cycle 1 increased under a boost of the control parameters 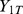 or 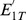. In fact, the growth of the intermediate complex 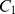 is compensated by a corresponding decrease in 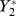, keeping a roughly constant total phosphorylated fraction 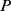. As before, to get a large variation of 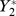 by making available more protein 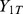, cycle 1 should be deactivated, leading to the phenomenon of total sequestration in a range of 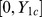 (Fig. 3(G)). In contrast, if the control parameter is the phosphatase of the downstream cycle, then a retroactive response on cycle 2 is possible if the downstream cycle starts activated, while 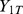 is large enough (cf. Fig. 3(I)).

### Retroactive Signaling in Multi-cycle Pathways

**Figure 4:**
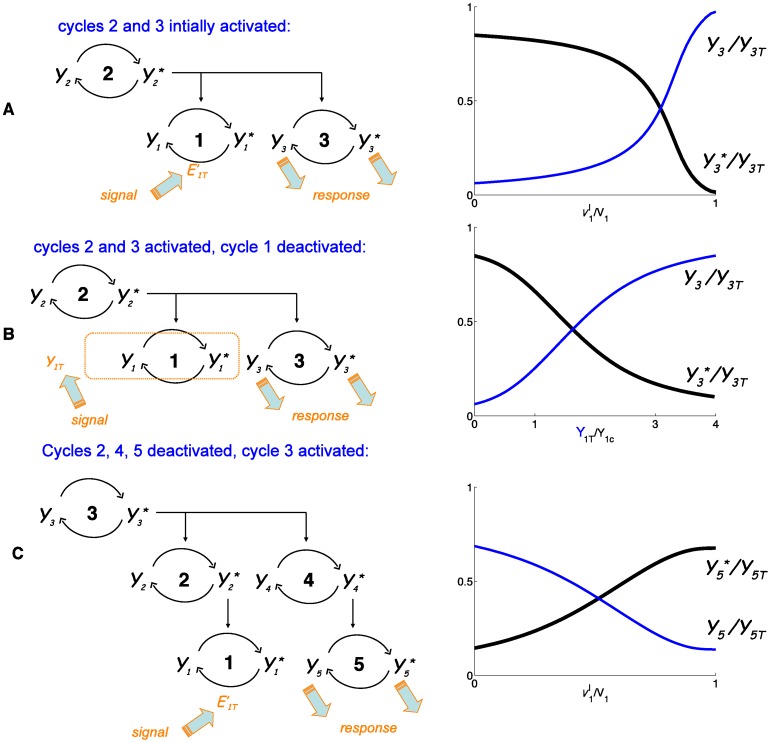
Retroactive signaling in multi-cycle pathways. 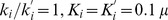
M for all 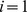 to 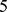, except for (A)–(C) 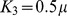
M, and for (D) 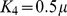
M. (A) 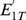 is varied in the range [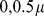
M] such that 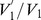 goes from 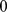 to 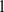. 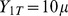
M, 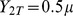
M, 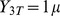
M, 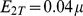
M
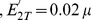
M, 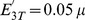
M. (B) same but 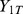 is varied on the range 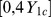 and 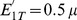
M. (C) identical to (A) except that cycle 2 is deactivated, with 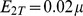
M
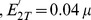
M. (D) 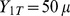
M, 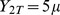
M, 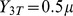
M, 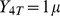
M, 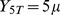
M, 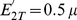
M, 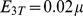
M
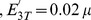
M, 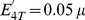
M
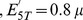
M.

The results obtained with a 2-cycle cascade can predict the effect of retroactivity in short signaling pathways with more than 2 cycles. We first consider a 3-cycle pathway where the activated protein in the cycle at the top of the pathway is an enzyme that activates two other cycles which are not directly linked together (Fig. 4(A)-(B)). In the last section we have demonstrated that a change in the parameters of a downstream cycle, for example the amount of phosphatase or the available protein of the cycle 1, can affect the state of the upstream cycle 2. More precisely, we anticipate that when the phosphatase is increased in cycle 1, it can augment the deactivated form of the protein 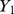. The latter then can bind to a greater amount of enzyme 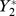, which become less available for the activation of other substrates such as the protein in cycle 3. Therefore, to implement the scheme of retroactive signaling 1
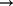
2
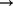
3, we start by assuming that the upstream cycle 2 is activated and we consider a signal having the form of an increase in the phosphatase of the downstream cycle 1. We know from the above results (cf. Fig. 3)(I)) that to create a substantial variation in the upstream cycle 2, the phosphatase signal should switch the cycle 1 from an activated state to a deactivated state, considering at the same time a relatively large amount of available protein in cycle 1 (cf. Eq.(6)). Then Fig. 3(I) showed that the switching of the downstream cycle caused a complete decrease of the free phosphorylated enzyme 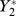 in the upstream cycle 2. This behavior of 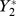 can be considered as an output response of the pathway 1
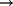
2 that can be used as the input of the conventional signaling pathway 2
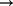
3. Therefore a retroactive signaling in the 3-cycle pathway 1
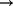
2
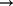
3 shown on Fig. 4 is promoted when there is a strong retroactivity on the segment 2
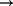
1, but a weak retroactivity on the segment 2
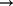
3 with respect to the considered input. Another condition is that, when the downstream cycle 1 is completely activated (i.e. when the phosphatase signal on cycle 1 is absent), cycle 3 should be activated by cycle 2. In this case only, it will feel the strong decay of the free phosphorylated enzyme in the upstream cycle 2 caused by its sequestration in the compounds of cycle 1. Figure 4(A) illustrates this type of signaling. One sees that cycle 3 can be switched on or off by varying the phosphatase regulating the input cycle 1.

A similar retroactive signaling in the same 3-cycle pathway can be achieved by modifying not the phosphatase but the available protein in the starting cycle 1. Keeping the same parameters as above, Fig. 4(B) shows that increasing the signaling protein 1 from a low value to four times the characteristic range 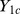 entails a deactivation cycle 3. This happens because of the retroactive mechanism between cycles 1 and 2, as discussed in the previous section (cf. Fig. 3)(G)). In the latter case, the increase of the total protein available in the downstream cycle 1 downregulated the activated enzyme in the upstream cycle 2, assuming that the downstream cycle was deactivated. Here again, by combining a large retroactivity between cycles 1 and 2, but a low one between cycle 2 and 3, one achieves a retroactive signaling between cycle 1 and 3.

In some covalent modification cycles, the deactivated protein can serve also as an enzyme for another protein modification [18], [20]. For example a variation of the motif shown on Fig. 4(A) is a 3-cycle network consisting of one upstream cycle and 2 downstream cycles activated respectively by the phosphorylated and non-phosphorylated forms of protein in the upstream cycle. Then we checked that a change in the phosphatase of one downstream cycle can produce a transition in the other downstream cycle activated by the non-phosphorylated protein in the upstream cycle (not shown).

To extend the possibility of retroactive signaling to more complex situations than a 3-cycle pathways we now consider a motif of a 5-cycle network in which the activated protein in the top cycle acts as the enzyme regulating two 2-cycle cascades, as shown on Fig. 4(C). Can we produce in this case an example of retroactive signaling from one bottom cycle to the other bottom one, numbered respectively by 1 and 5, initiated for instance by a phosphatase variation in cycle 1? Here, the study of the 2-cycle and the 3-cycle systems reported above can also help to answer this question. In this 5-cycle pathway, the subnetwork formed by cycles 2-3-4 has the same topology than the 3-cycle pathway discussed previously. Therefore, since this latter subsystem is suitable for retroactive signaling, let us consider the subnetwork 2-3-4 with the same parameters as considered for the 3-cycle network of Fig. 4(B). Then we can link to this system the cycle 1 downstream to cycle 2, and the cycle 5 downstream to cycle 4. For recall, cycle 2 is deactivated. Now we use the result shown on Fig. 3(H), showing that increasing the phosphatase in cycle 1 is going to reduce the available protein in cycle 2 in such a way that the free activated enzyme in cycle 3 is strongly reduced. This, in turn, deactivates cycle 4, and then cycle 5 as for standard cascades. This example of retroactive signaling scenario is seen on Fig. 4(C) where the increase in the phosphatase in cycle 1 entails not only the deactivation of cycle 1 (not shown) but also the deactivation of the remote cycle 5. Let us remark that this crosstalk effect can propagate to possible downstream effectors activated by cycle 5.

## Discussion

Cell signaling is generally thought in terms of a series of reversible biochemical reactions that are chained together in a feedforward network where extra connections, called feedbacks, could regulate the information flow from bottom-up. In particular the expression “signaling cascade” was coined to suggest the idea of an upstream to downstream signal transmission. In the simplest scheme of a cascade of two covalent modification cycles, the input signal typically is a steep increase of the enzyme modifying the first protein. Then the latter acts as the enzyme activating the second protein whose concentration is interpreted as the output of this system. In this paper, however, we show that in such a cascade a retroactive signaling is also possible, i.e. transmitting an input signal from downstream to upstream, and we predict conditions for which this phenomenon can be observed. The input signal is now a variation of a biochemical species that can change the state of the downstream cycle. Two cases are considered, namely a change of the total amount of the downstream signaling protein, or a variation of the phosphatase deactivating the same protein. In both cases we work out characteristic ranges of the concentrations of the species for which a retroactive effect can be observed in the upstream cycle. Moreover we show that this potentiality can help to perform retroactive signaling in short multi-cycle pathways.

A covalent modification cycle is generally described as a two-state entity for which the total level of protein is fixed. However, like all the molecules inside the cell, this signaling protein is subjected to a turnover governed by several processes, including synthesis and degradation [21]. The changes in these processes alters the total level of proteins. For example the degradation of several signaling proteins is actively regulated by proteases, which has consequences on the signaling dynamics [22]. The present study shows that the variation of the total amount of available protein in a downstream signaling cycle can also affect the states of signaling modules upstream in the transduction cascade.

There are several ways to modify the available protein in the downstream cycle in a cascade of covalent modifications. One way is to change the amount of substrates to which the activated protein of the downstream cycle can bind. For example, in a recent study reported in [17], the authors perform experiments on the ERK/MAPK pathway associated with the syncytium state of the *Drosophila* embryo. They manage to modify the amount of substrates of the doubly phosphorylated form of ERK by constructing mutants missing the corresponding substrates. Another way to alter the available protein in the downstream cycle is to add in the medium a kinase inhibitor that can bind to the activated enzyme at the end stage of the pathway [16], [23]. Both ways can be modeled by considering an additional chemical reaction of the form:
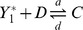
(7)where 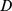 represents a substrate or a kinase inhibitor of the downstream protein 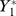. Then it can be shown that the set of stationary state equations of the signaling pathway is affected only in the conservation equation for the total protein 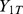. More precisely this latter quantity is replaced by 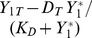, where 2 additional parameters characterize respectively the total amount 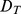 of binding chemical species and the dissociation constant 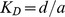. Thus, the effect of varying 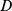 is qualitatively analog to changing the amount of available protein 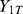. In particular, when the affinity of 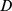 for protein 1 is high (i.e., 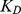 small), the available downstream protein is approximately reduced by 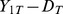. Therefore under this hypothesis the upstream response in a 2-cycle cascade to a variation of 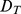 can straightforwardly be inferred from the curves shown on Figs. 3. For instance, from Fig. 3(B) one predicts that in a 2-stage cascade increasing 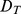 can decrease the phosphorylated fraction 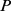 of the upstream protein, especially if the upstream cycle is in a deactivated state. This phenomenon may be the source of undesirable off-target effects in targeted therapies based on kinase inhibitors [16].

In Ossareh et al, the authors performed mathematical analysis of retroactivity in a signaling cascade with an arbitrary number of stages. They achieved necessary and sufficient conditions for which retroactivity exists in such chains. Their analysis is based on the linearization of the steady state equations in order to predict how a small downstream perturbation is amplified in the upstream response of an arbitrarily long signaling chain. Those results are complementary to the ones presented in the present paper, in the sense that here we consider short signaling pathways but our analysis is based on the resolution of the full nonlinear equations, and not only on the linearized system. So, it is concerned with arbitrarily large perturbations of the parameters. In fact we show that retroactive signaling is meant to work only for a characteristic range of parameter variations that we analytically estimate by working on the asymptotic behaviors of the system for small and large parameter perturbations.

Signaling pathways are regulated by several mechanisms, like positive or negative feedback loops linking the output of the cascades and some upstream stages. This requires the existence of specific chemical interactions between the output protein of the cascade and the upstream proteins that are involved in the feedback loop. Our study shows that the property of retroactive signaling can be another way to regulate the functioning of signaling cascades in branched pathways, without explicit feedbacks. In fact, we can further speculate that in natural signaling pathways with possibly several branches, some of the latter would be sensitive to retroactivity and be devoted to the regulation of the usual branches, where signals go in the top-down direction. These results prompt new experiments concerning signaling cascades and possibly new ways to interpret previous results.

## Methods

Our theoretical study is performed in the framework of coupled nonlinear equations describing the rate of changes of protein concentrations in signaling cascades formed of covalent modification cycles. The model equations are deterministic and based on the law of mass action. Only stationary states of these equations are analysed and thus the mathematical method amounts to solving sets of algebraic nonlinear equations. Thus the issue of how the biochemical species reach the equilibrium is not discussed here, as it has been addressed in some previous studies [8], [9], [24]. In this respect our analysis is independent of questions related to possible time-scale differences between the kinetics of enzyme/substrate. For example, the usual quasi-steady state approximations are not to be considered since all the variables are at equilibrium.

Let us note that we assume that the studied signaling pathways possess a stable equilibrium. Although in this paper we will not explicitly discuss the generality of this assumption by performing the linear stability analysis of the equation set, the hypothesis of a stable equilibrium is consistent with the current knowledge. In the literature, published results indicate that non steady behaviors (e.g. sustained oscillations) can arise in signaling cascades only with the concomitant occurence of bistability in the signaling modules [6], [25]. However, this situation was only met with signaling modules described by double-phosphorylations cycles, like in the MAPK cascade. Here the considered signaling pathways do not include double-phosphorylation. Therefore this paper will not consider retroactive signaling in oscillating systems.

### Steady States in Basic Models of Signaling Cascades

Let us introduce the notations used for writing the equations in the case of the simple 2-cycle cascade as depicted on Fig. 1(A). Assuming that this system is isolated from other biochemical reactions, the chemical equations describing the transformations of these species can be written as follows:
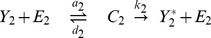


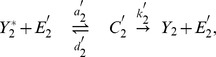


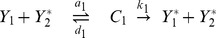


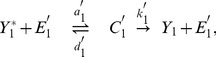
(8)where 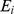 and 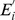 denote enzyme concentrations, whereas 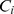 and 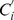 (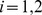) are intermediate enzyme-substrate complexes. These chemical equations readily generalize to the other motifs, e.g. the one shown on Fig. 1(B). The kinetic equations of the state variables of the cascades are written using the law of mass actions.



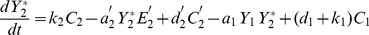
(9)

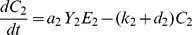


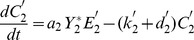


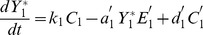
(10)

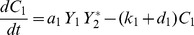


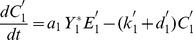
with the conservation laws for the total proteins 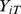 and total enzyme concentrations 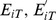:



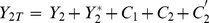
(11)

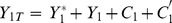
(12)

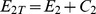


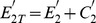


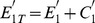
Since we focus only on the stationary states of the system, the time-derivatives of the concentrations can be equaled to zero. This enables to express the variables 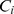 and 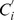 (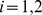) in terms of the protein concentrations as follows:



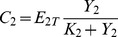


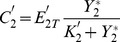


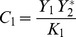
(13)

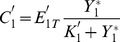
(14)with the coefficients 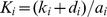 (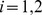) defined as a function of he kinetic parameters 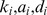. One thus recognizes the usual Michaelis-Menten form for the substrate-enzyme complexes. The substitution of these expressions in Eqs.(9)–(10) and in the conservation laws given Eqs.(11)–(12) leads finally to 4 algebraic equations in the unknowns 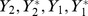. Therefore a reduced set of equations (9–14) can be written as:



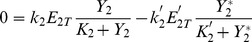
(15)

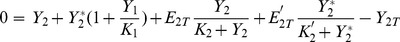
(16)

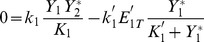
(17)

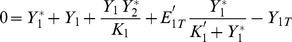
(18)


A 2-cycle cascade involves 4 enzymatic reactions. Each of those can be characterized also by their maximum reaction rates (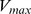). We denote the latter as follows:
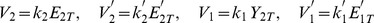
(19)The upper bound of the velocity 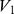, which describes the activation of 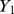, will depend on the total protein in cycle 2. In the following section we will seek the conditions under which the variations of parameters of cycle 1 produce a significant effect in cycle 2 due to retroactivity. As will be discussed, this property will depend on the states of the variables of both, upstream and downstream cycles. We will use the following terminology: cycle 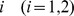 is said to be *activated* if 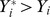. Otherwise, it is said to be *deactivated*. This property is easily related to the ratio 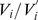 in the symmetric case 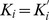. Then cycle 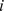 is activated if and only if 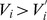 [5].

The following sections give details on the derivation of equations (1)–(3) and (5) used in the section Results.

### Variation of the Total Downstream Protein in a 2-cycle Cascade

Let us consider a 2-cycle cascade as drawn on Fig. 2(A), with total upstream protein 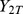, total downstream protein 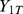, and total deactivating enzyme 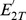 and 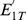, respectively for the upstream and downstream cycles. We wish to determine a suitable value of 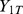 that can be used as a characteristic dose of downstream protein inducing a retroactive effect on the upstream cycle. The steady state of this system is given by the solution of Eqs.(15)–(18). As motivated above, we focus on the behavior of 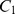, i.e. the intermediate substrate-kinase complex, which at equilibrium is given by 
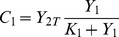
. The change of 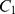 as a function of the total protein 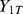 is illustrated on Fig. 2(B)–(C) in the case where 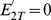, but the behavior is the same if 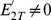. It can be sketched by an increase of 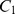 proportional to 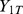 followed by a saturation to a constant value, that is 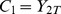 when cycle 1 is deactivated (i.e. 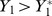). Therefore the quantity
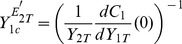
(20)defines a proper characteristic range of 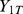 for the variation of 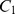. The upper index of 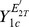 reminds that the result of the right-hand side of this equality depends on the value of 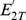. In particular, we will be interested to the case 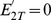 which corresponds to the situation of the isolated signaling cycle 1 with kinase 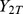 and with phosphatase 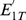. To simplify the notations, we will denote in the following:
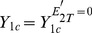
(21)and we will show that Eq.(1) holds with this definition. Since 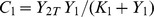, one deduces that



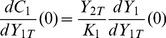
(22)Now, it suffices to compute the derivative of 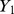 w.r.t. 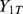 and evaluate it at 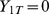. This can be analytically performed by differenciating each equation of the system (15)–(18) with respect to 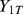. This calculation provides a system of linear equations in the coupled variables 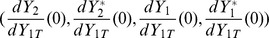. Solving this linear system we find that the solution can be written as:
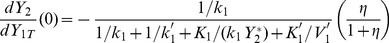
(23)

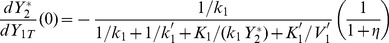
(24)

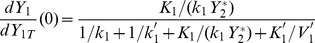
(25)

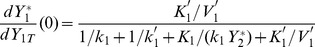
(26)where 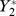 is the activated upstream enzyme when 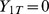, and 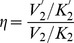. Let us remark that 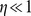 or 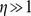 means respectively that the upstream cycle is highly activated or strongly deactivated.

By combining Eqs.(20), (22) and (25), one obtains the characteristic range for 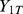, as defined by Eq.(20):
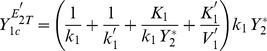
(27)In the case where 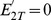, the upstream cycle is such that there is no phosphatase to deactivate it, so that 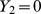 and 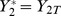. In this case, using the definition 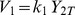, Eq.(27) becomes the sought relation Eq.(1), i.e.:
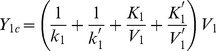
(28)One easily shows that 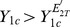 (because 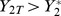). Therefore 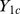 can be used as an upper bound of the characteristic range for 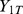. Particularly, if the downstream cycle is strongly activated, then 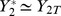 and then 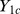 is an excellent approximation of 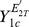. On the other hand, if the downstream cycle is strongly deactivated, so that 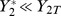, one can use 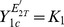, that is the lower value reached by 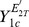 in the limit 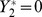.

Let us note that using the definition of 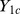 in the simple situation 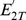, the derivatives 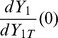 and 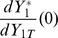 in eqs.(25)–(26) can be written in a compact form, namely:
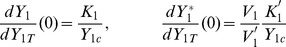
(29)Incidently, these expressions give the initial slope of the curves drawn on Figs. 2(D-E).

Now, to justify Eqs.(2)–(3) given in the Results, we wish to compute the asymptotic values of 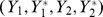 in the limit of large 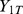. As suggested by the numerical computations, we first suppose that the asymptotic behavior of these variables are described by:



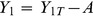
(30)

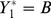
(31)

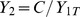
(32)

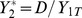
(33)where 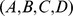 are unknown constants to be worked out. Substitution of these relations in Eqs.(15)–(18) with 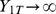 determines 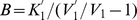. Since 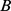 must be positive, this case is only consistent with the hypothesis 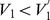, that is equivalent to Eq. (2) given in the Result section. Let us notice that here the result is independent on considering the case 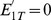 or not. The values of the other unknowns are found to be 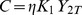, 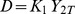, and



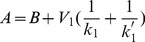
(34)Secondly, in order to justify Eq.(3), we suppose another asymptotic behavior for the system variables in the limit of large 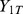:
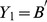
(35)

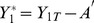
(36)

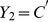
(37)

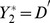
(38)where 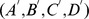 are new unknown constants to be determined. The calculation can be done in 2 steps. First 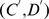 can be calculated by solving Eqs.(15)–(16) which here becomes:



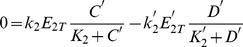


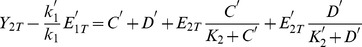
This system can be interpreted as finding the activated and deactivated proteins in the upstream cycle with the reduced amount of total protein 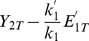. The latter must be positive, that is equivalent to the condition 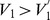 related to Eq.(3). The solution of this system is hard to write explicitly, except in the case 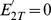 where 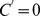 and 
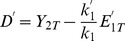
.

The second step is to solve Eqs.(17)–(18) in the limit 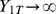. Then one easily finds that 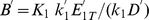, and therefore
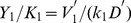
(39)where 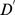 has been found in the first step. The latter equation generalizes Eq. (3), which holds in the case where 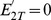. Then the simple expression of 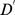 leads to the equality 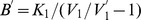 which is Eq. (3). Finally the value of 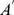 is the same expression as Eq.(34), but swapping the “primed” and “not primed” parameters.

In conclusion, by using Eqs.(29)–(38), let us note that we can sketch the behavior of 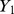 and of 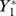 as a function of 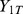 as piecewise linear graphs (see red lines on Figs. 2(D)–(E)).

### Variation of the Downstream Phosphatase in a 2-cycle Cascade

Let us consider a 2-cycle cascade as drawn on Fig. 1(A) and suppose now that the control parameter is the quantity of phosphatase 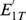 in the downstream cycle 1. We wish to prove the result of Eq.(5) giving the phosphorylated fraction 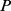 of protein in cycle 2 in the limit of large 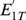.

First recall that 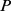 is defined by the chemical compounds containing 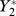, namely (cf. Eq.(4)):
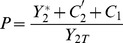
Thus, by using the steady expression for the complexes 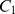 and 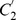, 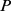 is also expressed as:
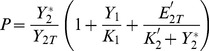
(40)We wish to remove the dependency in 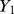 of this expression. The steady state equations of cycle 1 can be written as follows:



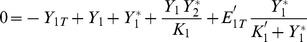
(41)

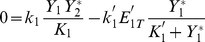



Since 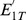 is an enzyme, in the limit 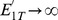, none of the biochemical variables should diverge. Therefore the second equation in the above system implies that in this limit one has 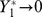. Thus the Eq.(41) can be simplified into the form:
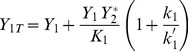
(42)This enables to write 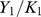 as:
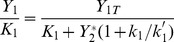
(43)And by using this expression in Eq.(40), one finds Eq.(5), or: 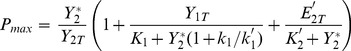
(44)

